# The impact of the 2020 diagnostic reference level revision and the development of national regulations on occupational radiation protection in medical facilities in Japan

**DOI:** 10.3389/fpubh.2025.1587287

**Published:** 2025-07-21

**Authors:** Takakiyo Tsujiguchi, Taito Ishikawa, Satoshi Arakida, Taiga Kanozawa, Teru Suzuki, Hajime Sakamoto, Takeshi Sasaki, Masataka Narita, Yasuyuki Takahashi

**Affiliations:** ^1^Hirosaki University Radiation Emergency Medicine and Cooperation Promotion Education Center for Disaster and Radiation Emergency Medicine, Hirosaki, Aomori, Japan; ^2^Graduate School of Health Sciences, Hirosaki University, Hirosaki, Aomori, Japan; ^3^Hirosaki University Hospital, Hirosaki, Aomori, Japan; ^4^Juntendo University, Tokyo, Japan; ^5^Ageo Central General Hospital, Ageo, Saitama, Japan

**Keywords:** radiation protection, occupational exposure, medical exposure, radiation safety management, diagnostic reference levels

## Abstract

This study examined the impact of revisions to the diagnostic reference levels and the Regulation on Prevention of Ionizing Radiation Hazards on radiation safety management in medical institutions by investigating changes in the awareness of occupational and medical radiation exposure and improvements in radiation protection. A self-administered questionnaire was sent to 799 facilities with angiography or nuclear medicine departments and responses were obtained from 424 facilities. The results showed that nonuniform exposure assessments were conducted in 93% of the facilities. Following regulatory revision, protective measures were reassessed in many facilities, leading to the increased use of protective eyewear and lens dosimeters. Notably, the adoption rate of lens dosimeters reached approximately 60%, suggesting heightened concerns about lens exposure. After the publication of the 2020 Diagnostic Reference Levels, many medical institutions reviewed the exposure conditions, with approximately 90% of the CT facilities modifying their parameters. These findings indicate that regulatory changes have contributed to an increased awareness of radiation protection and the reinforcement of specific protective measures.

## 1 Introduction

In 2011, the International Commission on Radiological Protection (ICRP) issued the “ICRP Statement on Tissue Reactions” (Seoul Statement) (1). This statement includes recommendations for the equivalent dose limit for the eye lens. Subsequently, the ICRP Publication 118 was published in 2012, providing a detailed explanation of its background and rationale (2). Furthermore, the recommendations were incorporated into GSR Part 3, issued in 2014 by the International Atomic Energy Agency, which established international safety standards (3). This development prompted the need to review the regulations on dose management for eye lenses in Japan. In 2017, the Radiation Council of the Nuclear Regulation Authority established the “Expert Committee on Radiation Protection for the Lens of the Eye.” This committee assessed lens exposure in Japan and examined specialized aspects of radiation protection, including appropriate measurement and evaluation methods (4, 5). In 2018, the “Expert Committee on the Revision of the Dose Limit for the Lens of the Eye” was established to support the necessary amendments to the Regulation on Prevention of Ionizing Radiation Hazards (RPIRH) regarding the dose limit for the lens of the eye. This marked the beginning of full-scale discussions about incorporating these regulations into Japanese law. In September 2019, the “Report on the Revision of the Dose Limit for the Lens of the Eye” was compiled (6). Subsequently, in 2021, the Medical Care Act and the Regulation on Prevention of Ionizing Radiation Hazards, a regulation concerning occupational safety for radiation workers, were amended. As a result, the dose limit for the eye lens for radiation workers was lowered from 150 mSv/year to 20 mSv/year, and the method for calculating and measuring the dose for the eye lens was partially revised (7, 8).

Diagnostic reference levels (DRLs) for medical radiation exposure were recently revised, and the DRLs 2020 was published (9). The number of radiology-based medical procedures in Japan has been increasing yearly (10). Additionally, radiation exposure during medical procedures is known to be higher than the average in other countries (11). This may have led to increased focus on reducing exposure levels in medical institutions.

Given the background described above, medical institutions across Japan are increasingly required to properly manage occupational and medical radiation exposure and reduce exposure levels to the minimum possible. Professional societies have also proposed various guidelines in this regard (12, 13). By contrast, according to a report from one of the largest companies handling personal dosimeters in Japan, approximately 0.5% of radiation workers, equivalent to about 1,000 individuals, encounter an equivalent dose exceeding 20 mSv/year on the eye lens (14).

In recent years, while radiation exposure management for both medical and occupational purposes has been discussed in the context of Japan, few comprehensive studies have investigated how specific medical institutions manage these issues and the types of awareness reforms implemented. Therefore, this study investigates how recent legislative revisions have impacted radiation workers’ awareness of occupational exposure and how the radiation protection environment has improved. By examining the status of radiation safety management in hospitals before and after regulatory revisions, this study provides insights that may be applicable to countries facing similar regulatory updates or seeking to enhance their radiation protection strategies. This study focuses on how Japan has implemented and adapted international recommendations on radiation protection—particularly concerning the dose limit for the lens of the eye—into its legal and medical systems. In Japan, the Radiation Council played a central role in regulatory reform, based in part on recommendations from the IAEA’s Integrated Regulatory Review Service. Unique aspects of the Japanese case include intensive discussions with medical professionals, the relatively high levels of medical radiation exposure compared to international averages, and ongoing challenges in occupational dose management. These circumstances highlight Japan as a distinctive case study of regulatory adaptation and professional awareness-raising. We believe that sharing the Japanese experience internationally can offer valuable insights for other countries facing similar regulatory changes or seeking to strengthen their radiation protection frameworks in clinical settings.

## 2 Methods

### 2.1 Outline of the questionnaire survey

A self-administered written questionnaire targeting the management of occupational and medical radiation exposure was sent to 799 hospitals in Japan that have more than 100 beds and are equipped with nuclear medicine departments and/or X-ray CT scanners. The questionnaire was completed by a representative from each institution (such as the chief radiological technologist). The response period was from July to August 2022. The specific contents of the questionnaire are shown in Table 1. The questions were designed to assess how radiation exposure management for radiation workers and medical exposure management in various departments has changed in response to the 2021 revision of the RPIRH and the release of DRLs 2020 in Japan.

**Table 1:** Questionnaire survey on occupational and medical exposure.

No.	Question content	Answer format	Answer options
1	What are the common body locations for wearing radiation dosimeters among hospital staff engaged in radiation-related work?	Single choice	□Head and neck □Thorax and abdomen □Both □Other
2	Are individuals exposed to uneven radiation doses during imaging or procedures that require wearing a lead apron?	Single choice	□Evaluating □Not evaluating
3	Are protective eyewear and lens dosimeters used as necessary?	Single choice	□Protective eyewear only □Crystal dosimeters only □Both are used □Neither is used
4	Have there been any changes in radiation protection methods before and after the revision of the Regulation on Prevention of Ionizing Radiation Hazards, which came into effect on April 1, 2021?	Single choice	□Changed since the amendment □Had changed since before the amendment □Currently under consideration □Other
5	Have there been any revisions to the exposure conditions for each modality in response to DRLs 2020? General X-ray examination Fluoroscopy Computed tomography Angiography Nuclear medicine	Single choice	□Yes □No □Other

### 2.2 Data analysis

The collected questionnaires were analyzed using simple tabulation, summarizing the management status of occupational and medical radiation exposure. The figures and tables were created using OriginPro 2020 (OriginLab).

### 2.3 Ethical consideration

The target medical institutions were informed of the purpose of the survey, voluntary nature of responses, protection of privacy, and anonymity in writing or by email before the survey was conducted. The name of the institution was left blank when responding to the questionnaire and consent to participate in this study was assumed to have been obtained upon submission of the questionnaire. This study was approved by the Ethics Committee of the Hirosaki University Graduate School of Health Sciences (approval number: 2021-030, approved on October 5, 2021).

## 3 Results

Valid responses were obtained from 424 institutions with a questionnaire response rate of 53.1%.

### 3.1 Management of occupational radiation exposure for radiation workers

**Figure 1:**
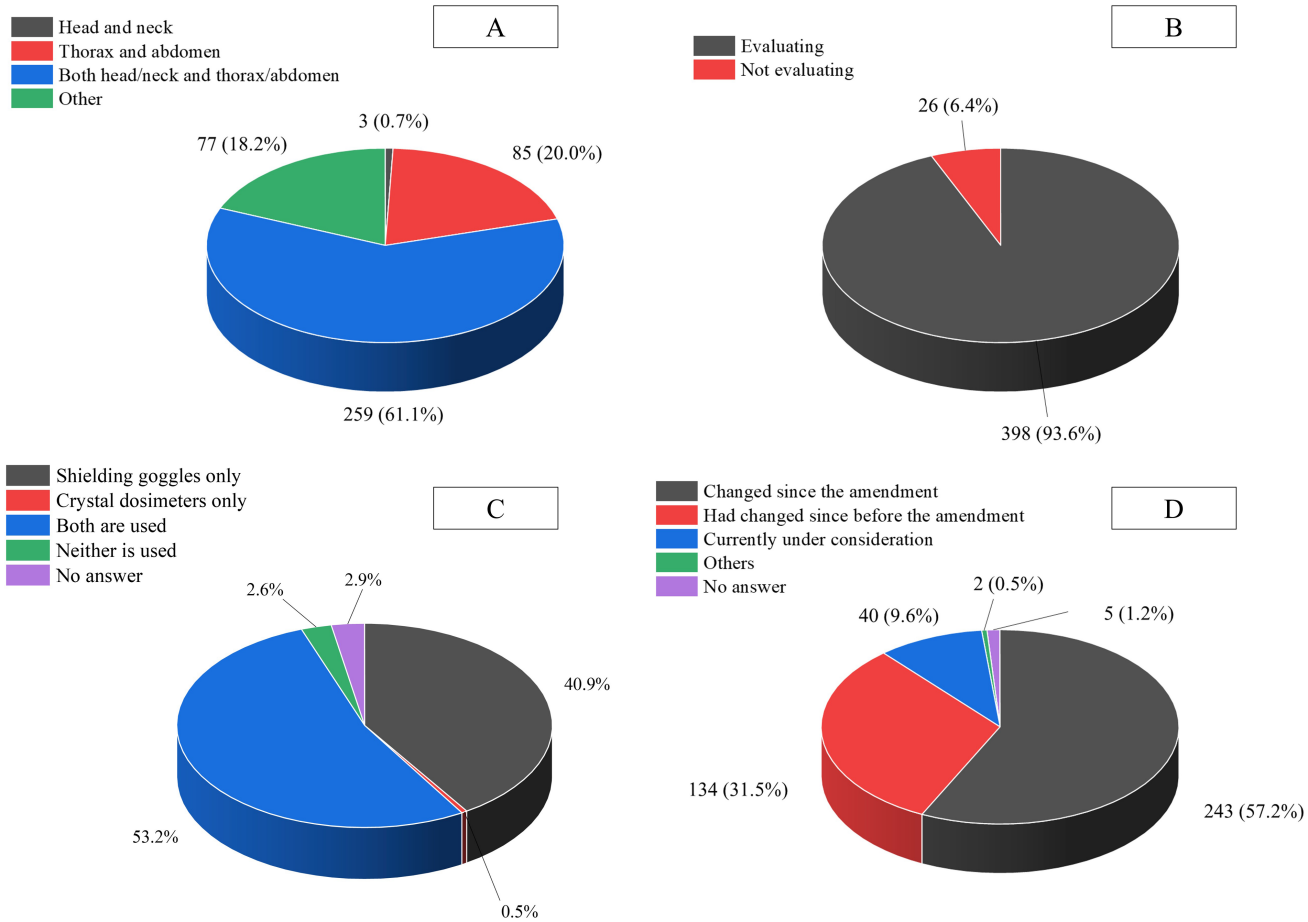
Results of the questionnaire survey. **(A)** Locations where radiation measuring devices are worn by staff engaged in radiation work within hospitals. **(B)** Whether or not evaluation of uneven radiation exposure is conducted. **(C)** The usage status of protective eyewear and crystal lens dosimeters. **(D)** Whether or not there were changes in protective measures following the 2021 amendment of the Regulation on Prevention of Ionizing Radiation Hazards.

Responses regarding the body sites where radiation workers in hospitals wore radiation dosimeters are shown in Figure 1A. Among the responding medical institutions, 61.1% reported that their radiation workers wore two or more dosimeters in total—one on the head and neck, and another on the thorax or abdomen. Other responses included institutions where workers wore dosimeters for specific purposes, such as ring dosimeters for hand exposure and dosimeters for eye lens monitoring. Additionally, there were no medical institutions that did not manage occupational exposure. The results of the questions regarding whether radiation exposure was predominantly from one side of the person, as well as the use of protective eyewear and lens dosimeters, are shown in Figures 1B,C. Geometric exposure condition was observed in 93.6% of the facilities, and more than half of the medical institutions used both protective eyewear and lens dosimeters. The responses to the question regarding the changes in protection methods after the revision of the RPIRH are shown in Figure 1D. Before the revision of the RPIRH, 378 facilities, accounting for approximately 88.7% of the total facilities, had already implemented protective measures. In addition, 42 facilities are in the process of making changes or planning to do so. Most facilities have reviewed their protection methods in response to the revised RPIRH.

### 3.2 Management of medical radiation exposure in relation to DRLs 2020

The responses to the question regarding the review of exposure conditions for each modality in response to the DRLs 2020 are shown in Table 2. In all modalities, more than half of the facilities reviewed their exposure conditions. In particular, 89.9% of the facilities reviewed their exposure conditions for X-ray CT examinations. In addition, many facilities indicated that they compared the published DRLs 2020 data with their own exposure conditions, and because there were no issues with radiation doses, they continued to operate without making changes.

**Table 2:** Whether or not exposure conditions have been revised for each modality in response to DRLs 2020.

Answer	Examination modality				
	General X-ray examination	Fluoroscopy	Computed tomography	Angiography	Nuclear medicine
Yes	237 (56.0%)	217 (51.2%)	381 (89.9%)	309 (72.9%)	295 (69.6%)
No	165 (39.0%)	191 (45.0%)	33 (7.8%)	92 (21.7%)	114 (26.9%)
Other	22 (5.2%)	16 (3.8%)	10 (2.4%)	23 (5.4%)	15 (3.5%)

## 4 Discussion

The response rate of 53.1% in this study is considered relatively high for a specialized and burdensome survey targeting medical institutions. Multiple follow-up contacts and clear communication of the survey’s significance likely contributed to obtaining responses from a wide range of regions and hospital sizes, which helps ensure the representativeness of the results. However, detailed attribute data on non-responding facilities were not available, so a direct comparison between respondents and non-respondents could not be performed. This limitation should be addressed in future studies, but the current findings are interpreted as having a reasonable degree of representativeness. The survey revealed that over 90% of facilities nationwide conducted evaluations of geometric exposure condition, indicating a high awareness of radiation dose reduction. Among these, 80% of the facilities used head dosimeters for the evaluation of geometric exposure condition, and it was found that 60% of these medical facilities use special dosimeters to measure the dose to the crystalline lens of the eye, which is worn inside protective eyewear, for their workers. Several reports have indicated that the correct use of protective eyewear is essential for reducing lens exposure to radiation (15, 16). In addition to recent research trends in the field of radiation protection, legal revisions concerning the equivalent dose limits for eye lenses have become a noteworthy issue, and there has been increasing interest, especially in the management of occupational radiation exposure, over the past few years.

According to a 2024 report by the Japan Gastroenterological Endoscopy Society, the usage rates of protective eyewear and lens dosimeters during fluoroscopy-guided endoscopic procedures were 31.4 and 38.4%, respectively. While this report addresses a specific clinical context and is not directly comparable to our nationwide survey, it provides useful insight into the current status of radiation protection practices in certain high-exposure settings (17). Compared to the results obtained in this survey, there was a noticeable difference in the usage rates of both protective eyewear and lens dosimeters. The disadvantages of wearing protective eyewear and radiation-protective clothing include discomfort owing to factors such as weight, sweating, and decreased work efficiency (18). The questionnaire in this study was completed by a representative from each institution (such as the chief radiological technologist), and the responses reflected the overall status of radiation protection practices at the institution, including those of physicians. The low usage rate of protective clothing among physicians may be due to factors such as discomfort or decreased work efficiency.

In the DRLs 2020, the definition of the standard body size was changed from 50–60 kg to 50–70 kg (9). Therefore, it was difficult to make direct comparisons. However, based on the results of this study, DRLs have contributed to the optimization process, and while some medical institutions may have increased the dose for optimization, it is highly likely that the DRLs have led to lower exposure levels for many examinations and imaging sites. In addition, many facilities that reported not having changed their exposure conditions were already operating at levels below those indicated in DRLs 2020. This suggests that DRLs 2020 have served as a reference for determining appropriate exposure conditions in many facilities.

This nationwide survey conducted at medical institutions suggests that there is a growing interest in radiation protection efforts related to the management and reduction of occupational and medical radiation exposure in medical institutions across Japan. Numerous guidelines, including the DRLs discussed and published by working groups organized by professional radiation-related societies, as well as various surveys on occupational radiation exposure and the management of medical radiation exposure, have been released by many academic societies and organizations. Recent studies have indicated that protective eyewear alone may be insufficient to protect against lens radiation exposure, and the development of new protective clothing is progressing daily (19). To reduce radiation exposure, appropriate protective measures must be implemented based on an understanding of the characteristics of the equipment used in each modality. Therefore, it is important to establish an educational system on radiation protection.

## Data Availability

The original contributions presented in the study are included in the article/supplementary material, further inquiries can be directed to the corresponding author.
